# Lewis, Fischer 344, and Sprague-Dawley Rats Display Differences in Lipid Peroxidation, Motor Recovery, and Rubrospinal Tract Preservation after Spinal Cord Injury

**DOI:** 10.3389/fneur.2015.00108

**Published:** 2015-05-15

**Authors:** Humberto Mestre, Manuel Ramirez, Elisa Garcia, Susana Martiñón, Yolanda Cruz, Maria G. Campos, Antonio Ibarra

**Affiliations:** ^1^Faculty of Health Sciences, Universidad Anahuac Mexico Norte, Mexico City, Mexico; ^2^CAMINA Project Research Center, Mexico City, Mexico; ^3^Pharmacology Medical Research Unit, National Medical Center “Century XXI”, IMSS, Mexico City, Mexico

**Keywords:** spinal cord injury, lipid peroxidation, Sprague-Dawley, Lewis, Fischer 344, strains, motor recovery, rubrospinal tract

## Abstract

The rat is the most common animal model for the preclinical validation of neuroprotective therapies in spinal cord injury (SCI). Lipid peroxidation (LP) is a hallmark of the damage triggered after SCI. Free radicals react with fatty acids causing cellular and membrane disruption. LP accounts for a considerable amount of neuronal cell death after SCI. To better understand the implications of inbred and outbred rat strain selection on preclinical SCI research, we evaluated LP after laminectomy sham surgery and a severe contusion of the T9 spinal cord in female Sprague-Dawley (SPD), Lewis (LEW), and Fischer 344 (F344) rats. Further analysis included locomotor recovery using the Basso, Beattie, and Bresnahan (BBB) scale and retrograde rubrospinal tract tracing. LEW had the highest levels of LP products 72 h after sham surgery and SCI, significantly different from both F344 and SPD. SPD rats had the fastest functional recovery and highest BBB scores; these were not significantly different to F344. However, LEW rats achieved the lowest BBB scores throughout the 2-month follow-up, yielding significant differences when compared to SPD and F344. To see if the improvement in locomotion was secondary to an increase in axon survival, we evaluated rubrospinal neurons (RSNs) via retrograde labeling of the rubrospinal tract and quantified cells at the red nuclei. The highest numbers of RSNs were observed in SPD rats then F344; the lowest counts were seen in LEW rats. The BBB scores significantly correlated with the amount of positively stained RSN in the red nuclei. It is critical to identify interstrain variations as a potential confound in preclinical research. Multi-strain validation of neuroprotective therapies may increase chances of successful translation.

## Introduction

Lipid peroxidation (LP) is perhaps one of the most important hallmarks of spinal cord injury (SCI) pathophysiology (1). After SCI, reactive oxygen species (ROS) are produced secondary to the initial injury [i.e., superoxide $\left({\text{O}}_{2}^{-}\right)$ and nitric oxide (NO)], and react with each other to form neurotoxic compounds [i.e., peroxynitirite (ONOO^-^)] (2). These highly volatile molecules attack polyunsaturated fatty acids resulting in the disruption of cell membrane (1). By-products of LP such as malondialdehyde (MDA) and 4-hydroxynonenal (4-HNE) bind to cellular proteins compromising structural and functional integrity (3). LP after SCI follows a biphasic pattern (4). The initial phase begins in the first hour following injury, peaking at around 4 h, and continues until the 12 h period where it reaches basal measures (5). The second peak begins 24 h after SCI and continues up to 5 days (120 h) before returning to normal levels (4).

The most widely used animals in SCI research are rats due to their adequate size for surgical procedures, availability of injury devices (New York University, Ohio State University, and Infinite Horizons Impactors), and reproducible clinical evaluations such as the Basso, Beattie, and Bresnahan (BBB) open-field locomotor recovery scale. Not to mention that these models are easily accessible and affordable for any research institution. Outbred rat strains like Sprague-Dawley, Wistar, and Long Evans are the most popular as opposed to inbred Lewis (LEW) and Fischer 344 (F344). Nonetheless the latter are still commonly used. Due to limited knowledge on interstrain variations, strain selection is mostly done by convenience and availability. This is undesirable as there are considerable differences in SCI outcome between rodent species (6), mice strains (7), rat strains (8), and even between rats of the same strain but different vendors (9). These dissimilarities suggest that they are not immediately comparable to each other, meaning that certain degenerative processes are going to account for more of the damage seen after injury as opposed to others. The unregulated use of strains in preclinical research may even result in the overestimation of specific mechanisms of pathogenesis, and if so, certain therapeutic agents will work in one strain but not so for the other. This can already been seen in studies that demonstrate a significant improvement after treatment with cyclosporine A or FK506 in a model of retinal ganglion cell axotomy in Fischer but not LEW rats (10). Therefore, it is critical to define the true implications of interstrain variations in SCI research.

To better understand this, we decided to evaluate one of the cornerstones of SCI pathophysiology (LP) in three commonly used rat strains in SCI research: Sprague-Dawley, LEW, and F344. To our knowledge, this is the first study evaluating LP in both outbred and inbred rat strains. LEW rats have significant differences in the hypothalamic-pituitary-adrenal (HPA) axis and are known to have an exacerbated response to neurotrauma (11). This strain is also susceptible to experimental allergic encephalomyelitis (EAE); thereby, the immune response developed after injury is, in fact, more intense and with stronger impact in the outcome of SCI (12). We hypothesized that LEW rats would have greater levels of LP after sham surgery, and SCI and this would equate into a worse functional recovery and decreased preservation of rubrospinal tract neurons.

## Materials and Methods

### Ethical statement

Efforts were made to minimize the number of animals used, as well as their suffering. The Institutional Review Board of the Universidad Anahuac Mexico Norte approved the study. All procedures were in accordance with the National Institutes of Health (US) Guide for the Care and Use of Laboratory Animals, Mexican Official Norm on Principles of Laboratory Animal Care, and the ARRIVE Guidelines.

### Experimental animals

Seventy-eight female, 12 ± 0.5-week-old LEW (190 ± 4 g), F344 (164 ± 5 g), or Sprague-Dawley (SPD; 220 ± 6 g) rats were supplied by the Animal Breeding Center of CAMINA Research Project. The rats were housed in individually ventilated temperature and humidity controlled cages (strain- and age-matched two per cage) with hardwood chip bedding in a 12 h light/dark cycle room. All animals had free access to food and water.

### Study design

Sample size for each experiment was calculated using an alpha of 0.05 and beta of 0.20. All animals were randomized to each experiment, and basal statistical analysis of weight and age yielded no statistical significance between experimental sets. The present work consisted of three experiments. The first one analyzed the amount of LP in the spinal cord (T9 level) of LEW, F344, or SPD rats (*n* = 8 per group) subjected to a laminectomy (sham-operated animals). The second experiment explored the levels of LP in another three groups (LEW, F344, and SPD; *n* = 8 per group) that were subjected to severe spinal cord contusion. In order to assess LP, animals from all groups were euthanized 72 h after sham operation or SCI. The third experiment analyzed motor recovery and axon survival of SCI rats. For this purpose, another three groups (LEW, F344, and SPD; *n* = 10 per group) were subjected to SCI. Motor recovery was evaluated weekly until they were euthanized 60 days later. Rubrospinal tract preservation was evaluated by measuring the number of labeled rubrospinal neurons at both red nuclei [rubrospinal neurons (RSNs); *n* = 4 per group].

### Spinal cord injury

Rats were subjected to a severe spinal cord contusion as previously described (13). Thirty minutes after an intramuscular injection of a mixture of ketamine (50 mg/kg; Probiomed, Mexico City, Mexico) and xylazine (10 mg/kg; Fort Dodge Laboratories, Fort Dodge, Iowa), a laminectomy of the T9 vertebrae was done on all rats. For SCI, a 10 g rod was dropped onto the exposed spinal cord from a height of 50 mm, using the NYU impactor (NYU, New York).

### Evaluation of lipid peroxidation

Seventy-two hours after sham operation or contusion, animals were anesthetized and euthanized by cardiac perfusion with saline solution. Afterwards, 1 cm of spinal cord at the T9 level was dissected from every rat to measure lipid-soluble fluorescent products (LFP), which provide an index of LP according to a previously described method (14). The spinal cord fragments were homogenized in 3 ml of saline solution (0.9% NaCl). One-milliliter aliquots of the homogenate were added to 4 ml of a chloroform-methanol mixture (2:1, v/v). After stirring, the mixture was cooled with ice for 30 min to allow phase separation. The fluorescence of the chloroform layer was measured in a Perkin-Elmer LS50B Luminescence spectrophotometer at 370 nm of excitation and 430 nm of emission wavelengths. The sensitivity of the spectrophotometer was adjusted to 150 fluorescence units with a quinine standard solution (0.1 mg/mL). Results were expressed as fluorescence units/g tissue and evaluated by a blinded observer. Statistical analysis of the weights of spinal cord wet tissue of all samples yielded no significant differences between groups and experiments.

### Assessment of motor recovery

Behavioral recovery was assessed every week using the BBB open-field test of locomotor ability during a 2-month follow-up period (15). Three separate blinded observers evaluated all animals, and the average of the three scores was used.

### Retrograde labeling of rubrospinal neurons

In order to evaluate RSNs, 60 days after injury animals were randomly selected (*n* = 4 per group) and reanaesthetized. Five microliters of 10% tetramethylrhodamine dextran dye (FluoroRuby; Molecular Probes, Eugene, OR, USA) in phosphate-buffered saline (PBS) were injected into both rubrospinal tracts of the proximal stump after a complete transection of the spinal cord below the site of contusion (T12). Five days later, the rats were decapitated and their brains were excised, processed, and cryosectioned. Every other section (20 μm thick) of the red nuclei – an average of 44 sections – was qualitatively examined by fluorescence and confocal microscopy. Photomicrographs from each section were quantitatively evaluated by using a computer image analysis system (Image-Pro Plus, Media Cybernetics, ML, USA). Only large well-stained cells with whole body labeling were counted from the magnocellular part of red nucleus. The total number of labeled cells was counted in every section from each brain. The number of labeled cells recorded for each brain is the sum of all the cells counted in each section. The number of labeled neurons in each rat is given by the average number of cells counted in its two red nuclei by a blinded investigator. RSNs are reported as the percentage from sham operated rats (four rats with no injury).

### Statistical analysis

Statistical analysis was done using the Prism 6 software (Prism 6.0, GraphPad Software Inc., San Diego, CA, USA). Data are expressed as mean ± SD, unless otherwise specified. All data sets were analyzed for normality using the D’Agostino-Pearson omnibus test and homogeneity of variances to define whether to use parametric or non-parametric tests. Measurements of LP after sham and SCI required a non-parametric Kruskal–Wallis test with a *post hoc* Dunn’s multiple comparisons test. Data for these experiments were displayed as box and whiskers plots with maximum 75th percentile, median 25th percentile, and minimum. Functional recovery was evaluated using two-way repeated measures ANOVA with Bonferroni’s multiple comparisons posttest. RSN axon survival was analyzed using a Kruskal–Wallis test with a *post hoc* Dunn’s multiple comparisons test. Correlation between BBB scores and RSNs was done using Pearson’s correlation coefficient. All exact *P*-values <0.05 were considered statistically significant.

## Results

### Lewis rats present higher levels of lipid peroxidation after sham surgery

**Figure 1:**
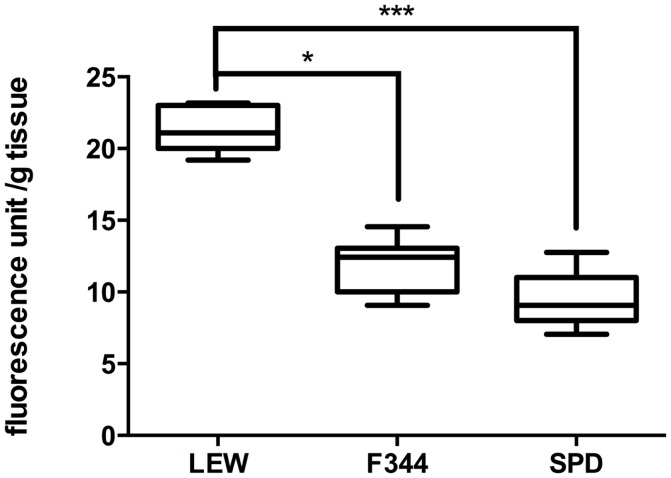
**Evaluation of lipid peroxidation in sham-operated rats**. LEW rats presented the highest levels of LP after laminectomy of T9. Boxes represent the distribution of the data (*n* = 8/group), extending from the 25th to the 75th percentiles; the line is drawn at the median; whiskers represent the highest and lowest values. *P < 0.05; ***P < 0.001. Kruskal–Wallis test with a *post hoc* Dunn’s multiple comparisons test.

Laminectomy by itself is an invasive procedure susceptible of inducing inflammation and release of free radicals. We evaluated the levels of LP in LEW, F344, and SPD rats subjected only to a laminectomy of the dorsal T9 vertebrae. Although LP products were barely detected in these rats, F344 and SPD rats (*n* = 8 per group) presented lower LP levels as compared to LEW. The amount of LP products observed in LEW animals (Figure 1: 21.4 ± 1.5 fluorescence unit/g tissue; mean ± SD) was significantly higher in comparison with either F344 (11.9 ± 1.9; *P* < 0.05 vs. LEW) or SPD rats (9.6 ± 1.9; *P* < 0.001 vs. LEW). There was no significant difference between SPD and F344 rats (*P* > 0.05). The results obtained in this experiment are in line with previously reported studies in our laboratory using the same technique to quantify LP after sham laminectomy (16). The LFP method was originally described by Triggs and Willmore (17), and has demonstrated to correlate closely with MDA quantification using the TBARS reaction (18). Our group has previously used this technique successfully, and corroborated its validity using a known lipid peroxidizing agent (ferrous sulfate) as an internal control (14).

### Lewis rats present significantly more lipid peroxidation after spinal cord injury

**Figure 2:**
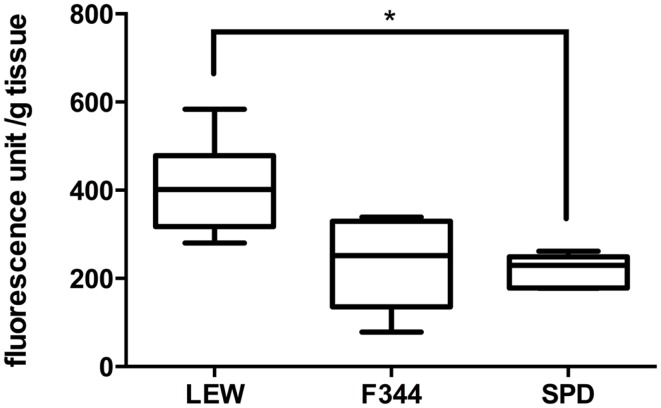
**Assessment of lipid peroxidation after severe SCI**. SCI significantly increased LP in LEW rats in comparison to SPD but not F344. Boxes represent the distribution of the data (*n* = 8/group), extending from the 25th to the 75th percentiles; the line is drawn at the median; whiskers represent the highest and lowest values. *P < 0.05. Kruskal–Wallis test with a *post hoc* Dunn’s multiple comparisons test.

After the evaluation of sham-operated rats, we proceeded to assess LP levels in spinal cord-injured animals (*n* = 8 per group). As can be seen in Figure 2, the levels of LP in LEW rats were significantly higher (407 ± 107 fluorescence unit/g tissue; mean ± SD) in comparison with SPD rats (220 ± 35; *P* < 0.05 vs. LEW) but not F344 (234 ± 103; *P* > 0.05 vs. LEW). LP was quite similar in F344 and SPD rats (*P* > 0.05*)*. The levels of LP seen in SPD rats are in accordance with prior studies that used the same injury paradigm and evaluation time period (16). Interestingly, there is a previous study that employed the same LP quantification method using a different injury model and follow-up period (24 h) in female Wistar rats (14). Although this time period is considered part of the first peak of LP and the data are not directly comparable to the present – due to varying methodologies – it may suggest that Wistar rats would also have LP levels similar to that seen in F344 and SPD.

### Motor recovery and retrograde RSN labeling were significantly lower in lewis rats

**Figure 3:**
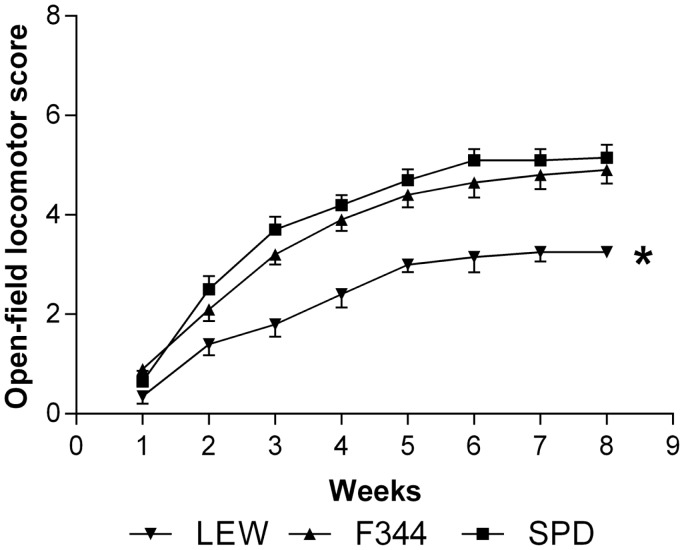
**Motor recovery of Lewis (LEW), Fischer 344 (F344), and Sprague-Dawley (SPD) rats subjected to SCI**. BBB score showed a significant improvement in F344 and SPD as compared to LEW rats. *P < 0.001. Repeated measures two-way ANOVA with Bonferroni’s *post hoc* test. Data of 10 rats per group.

**Figure 4:**
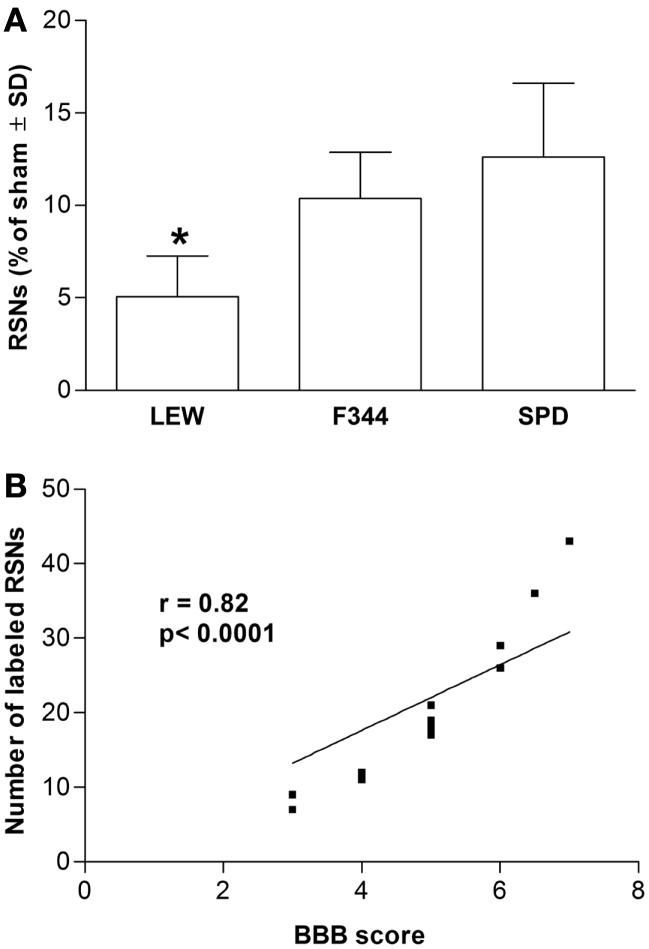
**Retrograde labeling of rubrospinal neurons in Lewis (LEW), Fischer 344 (F344), or Sprague-Dawley (SPD) rats with SCI**. **(A)** *Different from SPD (*P* < 0.05; Kruskal–Wallis test with a *post hoc* Dunn’s multiple comparisons test). Data represented as mean ± SD of four randomly selected rats (*n* = 4 per group). **(B)** This result significantly correlated with the BBB scores of assessed animals (*r* = 0.82, *P* < 0.0001, Pearson’s correlation coefficient).

**Figure 5:**
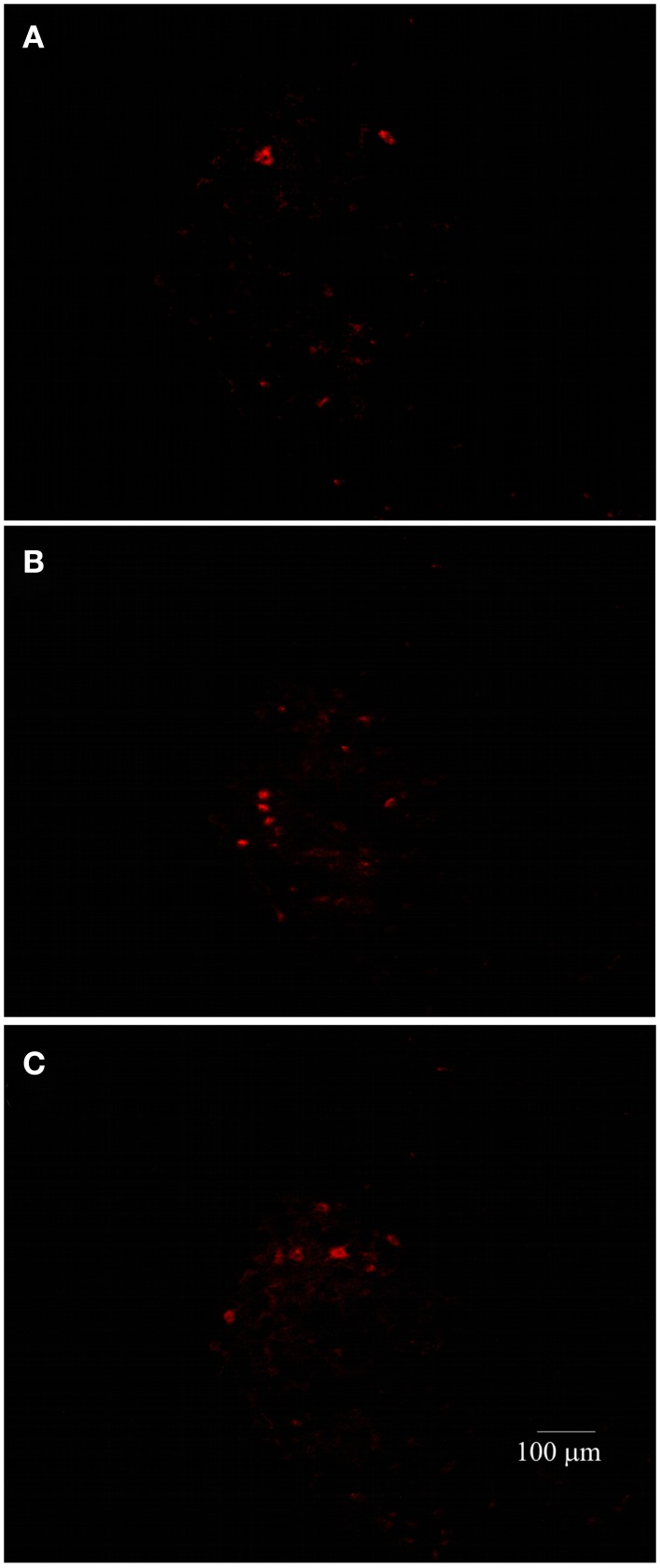
**Representative photomicrographs of the red nucleus of SC-injured Lewis (A), Fischer 344 (B), or Sprague–Dawley (C) rats**. The number of RSNs was lower in Lewis animals.

As LP is one of the most harmful events participating in tissue destruction after SC injury, we hypothesized that LEW rats would also present the lowest motor recovery and rubrospinal tract preservation after SCI. To evaluate this hypothesis, we subjected three groups of rats to spinal cord contusion (LEW, F344, and SPD rats). SCI rats were assessed weekly for motor recovery using the BBB scale. As was expected, LEW rats showed the lowest motor performance (Figure 3). Toward the end of the study, the BBB score of LEW rats was significantly lower (3.2 ± 0.3; mean ± SD) than the one showed by F344 (4.9 ± 0.8) or SPD animals (5.1 ± 0.7; *P* < 0.001, repeated measures two-way ANOVA followed by Bonferroni’s test). BBB score was not significantly different between F344 and SPD rats. To analyze tissue preservation, we then evaluated the number of preserved axons of the rubrospinal tract. The rubrospinal tract is more important in lower phylogenetic species that use limbs for locomotion (19, 20). The number of retrogradely labeled RSNs correlates with motor function (assessed by the inclined plane and Tarlov methods) in rats better than the corticospinal tract (21). Aside from this, previous studies in our laboratory have shown that BBB score provides reliable and consistent correlation with the number of labeled RSNs (22–26). We barely detected RSNs in LEW rats; they showed a mean of 12 ± 5 cells (5.05 ± 2% from sham-operated rats), while F344 and SPD showed 30 ± 7 (10.4 ± 2%) and 38 ± 5 cells (12.6 ± 4%), respectively (Figures 4A and 5). However, tract preservation was only significant between SPD and LEW (*P* < 0.05). There was no significant difference between F344 and SDP rats (*P* > 0.05). The total number of labeled red nuclei cells positively correlated with final BBB scores (*r* = 0.82, *P* < 0.0001, Pearson’s correlation) (Figure 4B).”

## Discussion

Lipid peroxidation is one of the most important and damaging effects of oxidative stress following SCI. The present study shows that LEW rats have significantly more LP after sham surgery and SCI than SPD and F344 rats. This in turn most likely reduces the preservation of the rubrospinal tract neurons resulting in a worse functional outcome after SCI. The 72 h time point was chosen in order to better evaluate the second LP peak. This initial phase of LP is caused by excitotoxicity (15 min to 6 h) and ischemia (1 h to 12 h) (4). On the other hand, the second peak is generated by neutrophil activation of microglia, monocytes, and activated macrophages that arrive into the CNS after injury (4). This increase in LP begins at 24 h and can last up to 120 h (4). Our results are in line with previous studies that demonstrate that LEW rats have elevated numbers of circulating peripheral blood mononuclear cells (PBMC), especially CD4^+^ and CD8^+^ T cells relative to F344 (27). These cells also have shown to have increased reactivity to lymphocytic mitogens such as concanavalin A (27). LEW rats have a more robust and lasting presence of activated macrophages and microglial cells at the site of injury after SCI than SPD (28). T-lymphocyte infiltration was also elevated twofold in LEW as compared to SPD by 72 h after SCI (28). It is also well known that LEW rats are susceptible to EAE, a rodent model of multiple sclerosis. Genomic studies have implicated the major histocompatibility complex (MHC) class II gene on chromosome 20, specifically RT1-Db1, in LEW rat’s predisposition to EAE (29). This MHC-II variant has a high affinity toward protein constituents of myelin, and therefore exacerbates the immune response after SCI (30).

The biological basis for the above mentioned variations could partially be due to significant differences in the HPA axis. All three strains have a pulsatile secretion of corticosterone, with one pulse occurring between 05:00 and 11:00 h and the second between 17:00 and 23:00 h (31). In LEW and SPD, mean circulating corticosterone levels are highest in the beginning of the dark cycle and lowest at the beginning of the light cycle, demarcating a clear circadian variation both in pulse frequency and amplitude (31). Conversely, F344 have no significant variations in either pulse frequency or height over a 24 h period resulting in relatively constant circulating corticosterone levels throughout the day (31). Lack of early morning nadir in F344 yields higher mean daily corticosterone levels significantly different to LEW and SPD (11). Corticotrophin releasing factor (CRF)-induced corticosterone levels rise rapidly in F344, peaking in 10 min compared to 30 min in LEW, suggesting a greater pituitary sensitivity in F344 (31). After exposure to a stressful stimulus (white noise), corticosterone levels fell rapidly in LEW but remained maximally elevated for 20 min in F344 (31). This posits that F344 are more insensitive to the corticosterone negative feedback mechanism making them hyperresponsive to stressful stimuli. These changes are also sex-dependent, with male LEW rats having lower circulating coricosterone levels than females and therefore higher PBMCs relative to females (27). The implications of sexual dimorphism on LP after SCI should also be studied in the future. There are differences in both central and pituitary control of the HPA axis in all three strains.

After SCI, immune cells produce the highest concentrations of ROS and in turn LP (16). Inbred strains, especially LEW demonstrate the highest levels of T cells and circulating PBMCs, together with a low concentration of corticosterone provides the ideal milieu to exacerbate LP. It is possible that F344 rats are protected from this response by having higher corticosterone concentrations and a faster and sustained response to the stressful stimulus (surgical procedure and SCI). This could possibly explain the differences in LP levels, decreased numbers of stained RSNs, and worse motor outcome in LEW as opposed to SPD and F344. These results beg the reappraisal of rat strain selection in confirmatory preclinical validation of neuroprotective therapies.

This work is not the first to establish interstrain variations after SCI. Previous works demonstrated that SPD rats (when compared to Wistar and Long Evans) recover faster from SCI and achieve a greater functional outcome (8). The gene expression pattern after SCI also varies greatly between strains, as seen in a study where SPD rats subjected to SCI have a considerable increase in the expression of genes associated with myelin structural proteins, while LEW showed no change; this gene upregulation improved BBB recovery in SPD rats (32). Interstrain variation is further complicated by intrastrain discrepancy. A recent study elucidated that SPD substrains from different vendors (Scanbur, Charles River, or Harlan) presented significant differences in locomotor recovery after SCI (9). The Harlan breeder substrain regained more hindlimb function than Charles River and Scanbur rats. These results are worrying, since they further opaque the true value of interlaboratory comparisons.

It would be worthwhile to take advantage of these differences in preclinical research. Interventions that are capable of inducing a statistically significant recovery after SCI in one outbred (Sprague-Dawley, Wistar, or Long Evans) and one inbred strain (Lewis) would be more likely to cross the translational barrier. However, the true potential of multi-strain validation for small animal (rodent) confirmatory preclinical research must be scrutinized further.

The present results demonstrate that LEW rats have significantly more LP after sham surgery and SCI than SPD and F344 rats. This consequently thwarts locomotor recovery possibly due to the significant death of rubrospinal tract axons. In SPD, F344, and LEW animals, the improvement in BBB score positively correlated with an increase in labeled RSNs at the red nuclei. Heterogeneity between strains may reflect interpatient variation seen throughout clinical trials. Therefore, it is possible that multi-strain validation of neuroprotective therapies may increase its chances of success through translation. In conclusion, an initiative should be made to (1) understand the true implications of rat strain selection in SCI research, (2) evaluate the utility of interstrain variations to improve translational research, and (3) the accuracy of making interlaboratory comparisons of interventional preclinical data when different rat strains are used despite identical study design.

## Conflict of Interest Statement

The authors declare that the research was conducted in the absence of any commercial or financial relationships that could be construed as a potential conflict of interest.
